# A Novel Prognostic Signature of comprising Nine NK Cell signatures Based on Both Bulk RNA Sequencing and Single-Cell RNA Sequencing for Hepatocellular Carcinoma

**DOI:** 10.7150/jca.85873

**Published:** 2023-07-16

**Authors:** Qi Yu, Xuefeng Shi, Hongjian Wang, Shun Zhang, Songnian Hu, Ting Cai

**Affiliations:** 1 Department of Experimental Medical Science, Ningbo No.2 Hospital, Ningbo 315010, China.; 2 Ningbo Institute of Life and Health Industry, University of Chinese Academy of Sciences, Ningbo 315032, China.; 3 State Key Laboratory of Microbial Resources, Institute of Microbiology, Chinese Academy of Sciences, Beijing 100101, China.; 4 College of Agricultural, Consumer and Environmental Sciences, University of Illinois at Urbana-Champaign, Champaign 61820, USA.; 5 Key Laboratory of Diagnosis and Treatment of Digestive System Tumors of Zhejiang Province, Ningbo 315010, China.; 6 Department of Pulmonary and Critical Care Medicine, Qinghai provincial people's hospital, Xining 81000, China.

**Keywords:** hepatocellular carcinoma, scRNA-seq, NK cells, 9-gene signature, prognosis

## Abstract

**Background:** Hepatocellular carcinoma (HCC) has limited prognostic prediction due to its heterogeneity. Understanding the role of natural killer (NK) cells in HCC is vital for prognosis and immunotherapy guidance. We aimed to identify NK cell marker genes through scRNA-seq and develop a prognostic signature for HCC.

**Methods:** We analyzed scRNA-seq data (GSE149614) from 10 patients and bulk RNA-seq data from 786 patients with clinicopathological information. NK cell marker genes were identified using clustering and marker finding functions. A predictive risk signature was constructed using LASSO-COX algorithm. Functional annotations and immune cell infiltration analysis were performed, and the nomogram's performance was evaluated.

**Results:** We identified 79 NK cell marker genes associated with NK cell-mediated cytotoxicity, apoptosis, and immune response. The multigene signature significantly correlated with overall survival (OS) in TCGA-LIHC cohort and was validated in other cohorts. Low-risk patients exhibited higher immune cell infiltration, including CD8^+^ T cells. The risk signature was an independent prognostic factor for OS (HR > 1, *p* < 0.001). The nomogram combining the risk signature and clinical predictors demonstrated robust prognostic ability.

**Conclusion:** We developed a nine-gene signature prognostic model based on NK cell marker genes to accurately assess the prognostic risk of HCC. This model can be a valuable tool for personalized evaluation post-surgery. Our study underscores the potential of NK cells in HCC prognosis and highlights the importance of scRNA-seq analysis in identifying prognostic markers.

## Introduction

Globally, primary liver cancer is the second most common cause of cancer-related death, with approximately 9.1% of cancer-related deaths attributed to it. Within primary liver cancer, hepatocellular carcinoma (HCC) accounts for 70%-90% of cases 1, 2. HCC is characterized by its aggressiveness and high resistance to chemotherapy and radiation, as well as a high rate of recurrence within five years following surgical treatment, occurring in approximately 60% of cases 3, 4. Although several RNA-sequence based prediction models are available for estimating patient prognosis and survival 5, they inadequately reflect the heterogeneity and tumor microenvironment of highly heterogeneous tumor like HCC. Thus, it is crucial to develop new predictive models and identify novel biomarkers to accurately forecast the prognosis and treatment efficacy in HCC patients.

Studies conducted recently have indicated that various components of the tumor microenvironment (TME), such as immune cells and inflammatory cells, are associated with tumor initiation and progression 6, 7. Consequently, alterations in the TME can have an impact on patient prognosis, making it essential to identify TME biomarkers for predicting patient outcomes 8, 9. In hepatocellular carcinoma, the TME is complex and comprises different components, including immune cells, stromal cells, cytokines, and extracellular matrix. Among the TME components, natural killer (NK) cells are critical in immune defense mechanisms.

They recognize and eliminate abnormal cells via MHC class I molecules and perforin/granzyme-mediated apoptosis and facilitate linking of innate and adaptive immune responses by producing cytokines and chemokines 10-12. Although the liver contains a higher proportion of NK cells than other organs 13, cytokines or other molecules released by cancer-associated fibroblasts (CAFs) or CD4^+^ T lymphocytes in the TME of HCC can inhibit NK cell activity, leading to HCC development 14, 15. Previous studies have also shown that the frequency of NK cells, activated killer cells, and interferon (IFN) production is significantly reduced in HCC 16. In HCC patients, an inverse relationship between HCC progression and NK cell activity has been observed 17, 18. Despite some studies on NK cell molecular features in liver cancer, their comprehensive analysis in the context of HCC is limited.

The technique of single-cell mRNA sequencing (scRNA-seq) has become a valuable tool for high-throughput and high-resolution transcriptome analysis at the cellular level, offering an excellent approach for studying tissue heterogeneity 19-21. Previous studies have shown the presence of a diverse EPCAM^+^ cell population in the liver that has the ability to produce bipotent liver organoids 22. In HCC patients, research on the T-cell landscape has identified the suppressive function of LAYN in both T-regulatory and exhausted CD8^+^ T-cells 23. Additionally, tumor tissue has been found to contain CD4/CD8 double-positive T (DPT) cells, which may originate from infiltrating CD4^+^ or CD8^+^ single-positive (SPT) cells 24. scRNA-seq has significantly improved our understanding of the TME and has the potential to predict patient prognosis 25, 26. For example, scRNA-seq analysis has uncovered the role of the lncRNA HCG18 in regulating tumor stem cells and macrophages, which can promote vascular invasion in HCC 27. Similarly, the development of a macrophage marker gene signature (MMGS) in breast cancer through scRNA-seq analysis has provided a valuable tool for predicting the antitumor immune response 28. Overall, these findings demonstrate the potential of scRNA-seq-based marker gene analysis for identifying novel immunotherapy targets and predicting patient outcomes in HCC.

The main aim of this study is to utilize scRNA-seq technology to uncover the molecular characteristics of NK cells in hepatocellular carcinoma (HCC) patients and develop a predictive model for clinical prognosis. We conducted a comprehensive analysis of scRNA-seq data from the GSE149614 dataset to identify NK cell marker genes and construct a gene signature. Using bulk RNA-seq analysis, we applied this signature to predict HCC patient prognosis. Validation was performed on independent cohorts from the ICGC-LIRI-JP and Gene Expression Omnibus (GEO) database. Univariate and multivariate COX regression analyses confirmed the independent prognostic significance of the NK cell marker gene signature. Integration of the signature with clinical risk factors resulted in a prognostic model capable of accurately predicting survival probabilities at different time points. These findings provide a valuable tool for guiding personalized treatment strategies in HCC.

## Materials and Methods

### Data Collection and Preprocessing

Totally, 804 samples were enrolled in this study, including 18 samples for scRNA-seq data 29, 362 samples for bulk RNA-seq data from The Cancer Genome Atlas (TCGA), 203 samples for bulk RNA-seq data from the International Cancer Genome Consortium (ICGC), and 221 samples for microarray data from the GEO database. The scRNA-seq data from 10 primary tumor samples and 8 adjacent normal samples of GSE149614 dataset were obtained from the GEO database and were used to identify NK cell marker genes. The scRNA-seq data were preprocessed using “Seurat” R package 30. The original data contained a total of 25,712 genes and 63,101 cells. The percentage of mitochondria and gene expression was calculated through the “Percentage Feature Set” function, and cells expressing less than 200 genes and more than 5% mitochondrial content were removed, resulting in 47,640 cells for further analysis.

To identify genes associated with survival and construct prognostic features, we collected a substantial amount of tumor transcriptomic data and clinical information from multiple sources. From the TCGA database, we retrieved data and clinical information of 362 patients through the UCSC Xena browser (https://xenabrowser.net/). For validation purposes, we acquired data and clinical information from the Hepatocellular Carcinoma Gene Expression Database (HCCDB) (http://lifeome.net/database/hccdb/home.html) for two additional cohorts: ICGC-LIRI-JP (n = 203) and GSE14520 (n = 221). The clinical information encompassed variables such as age, gender, family history, tumor stage, AFP, grade, and vascular invasion. A summary of the clinical data for the three cohorts can be found in Table 1. It is important to note that the datasets utilized in this study were obtained from publicly accessible repositories, and the original studies underwent rigorous scrutiny and received prior ethical approval.

### Single-cell RNA Sequencing Data Analysis

The scRNA-seq data was normalized through “Log Normalize” method, followed by identifying the top 2000 highly variable genes using the “Find Variable Features” function of the “Seurat” R package. Next, we scaled all genes using the "Scale Data" function and reduced the dimensionality of the data by performing principal component analysis (PCA) on the top 2000 highly variable genes using the "Run PCA" function. Subsequently, we employed the "Find Neighbors" and "Find Clusters" functions (with a resolution of 0.5) to cluster the cells. To visualize the clustering results, we used the "Run TSNE" function to generate a two-dimensional t-distributed stochastic neighbor embedding (t-SNE) plot. To identify differentially expressed genes (DEGs) for each cluster, we employed the "Find All Markers" function. For cell type annotation, we performed reference-based annotation using the reference data from the Cell Marker 2.0 31 and the Human Protein Atlas 32. We then used the "Subset" function to extract the T and NK cells population. For identification NK cell marker genes, we used a threshold of adjusted *p*-value < 0.01 and | log2 (fold change) | > 0.5.

### LASSO-COX Dimension Reduction Analysis

LASSO-COX dimension reduction analysis was performed using the “glmnet” and “survival” R packages. We performed a 10-fold cross-validation using the "cv.glmnet" function to determine the optimal model. The tuning parameter lambda was chosen based on lambda min. Consequently, we obtained a list of 9 candidate genes with non-zero beta coefficients: FABP5 (0.051464303), GZMH (-0.021906317), RAP1B (0.070662833), APOC1 (-0.003715174), PKM (0.051620391), EIF4A3 (0.128769934), RGCC (-0.054564185), IL7R (-0.068407441), and FTH1 (0.040192974). To calculate the risk score for each patient, we utilized the following formula: Risk score = (0.051 × FABP5 expression) + (-0.022 × GZMH expression) + (0.071 × RAP1B expression) + (-0.004 × APOC1 expression) + (0.052 × PKM expression) + (0.129 × EIF4A3 expression) + (-0.055 × RGCC expression) + (-0.069 × IL7R expression) + (0.040 × FTH1 expression).

### Estimation of Stromal and Immune Score

The ESTIMATE algorithm 33, implemented through the “estimate” R package, was utilized to evaluate the extent of stromal and immune cell infiltration in the TCGA cohort. The stromal score, immune score, ESTIMATE score, and tumor purity score were compute. We used CIBERSORT analysis to compare differences in various immune cells in distinct groups 34.

### Nomogram Construction

The nomogram was developed using the “rms” R package in the training set. The scoring system was represented in the upper part, while the lower part depicted the prediction system. By calculating the cumulative score based on individual factors, the 1-, 2-, 3-, and 5-year survival rate of HCC patients could be accurately predicted. The area under curve (AUC) of the receiver operating characteristic (ROC) curves, calibrate curves and C-Index values were used to show the accuracy of the survival prediction.

### Statistical Analysis

Statistical analyses were performed using R software (version 3.6.0, https://cran.r-project.org/) and SPSS statistics software (version 25.0, IBM). The prognostic significance was assessed using Kaplan-Meier curves analysis and COX analysis. Functional enrichment analyses, including Gene Ontology (GO) and Kyoto Encyclopedia of Genes and Genomes (KEGG), were conducted using the Database for Annotation, Visualization and Integrated Discovery (DAVID) portal website (https://david.ncifcrf.gov/tools.jsp). The threshold for statistical significance was set as *p* < 0.05.

## Results

### Definition of Cell Compositions from HCC Patients' Liver Tissues

**Figure 1:**
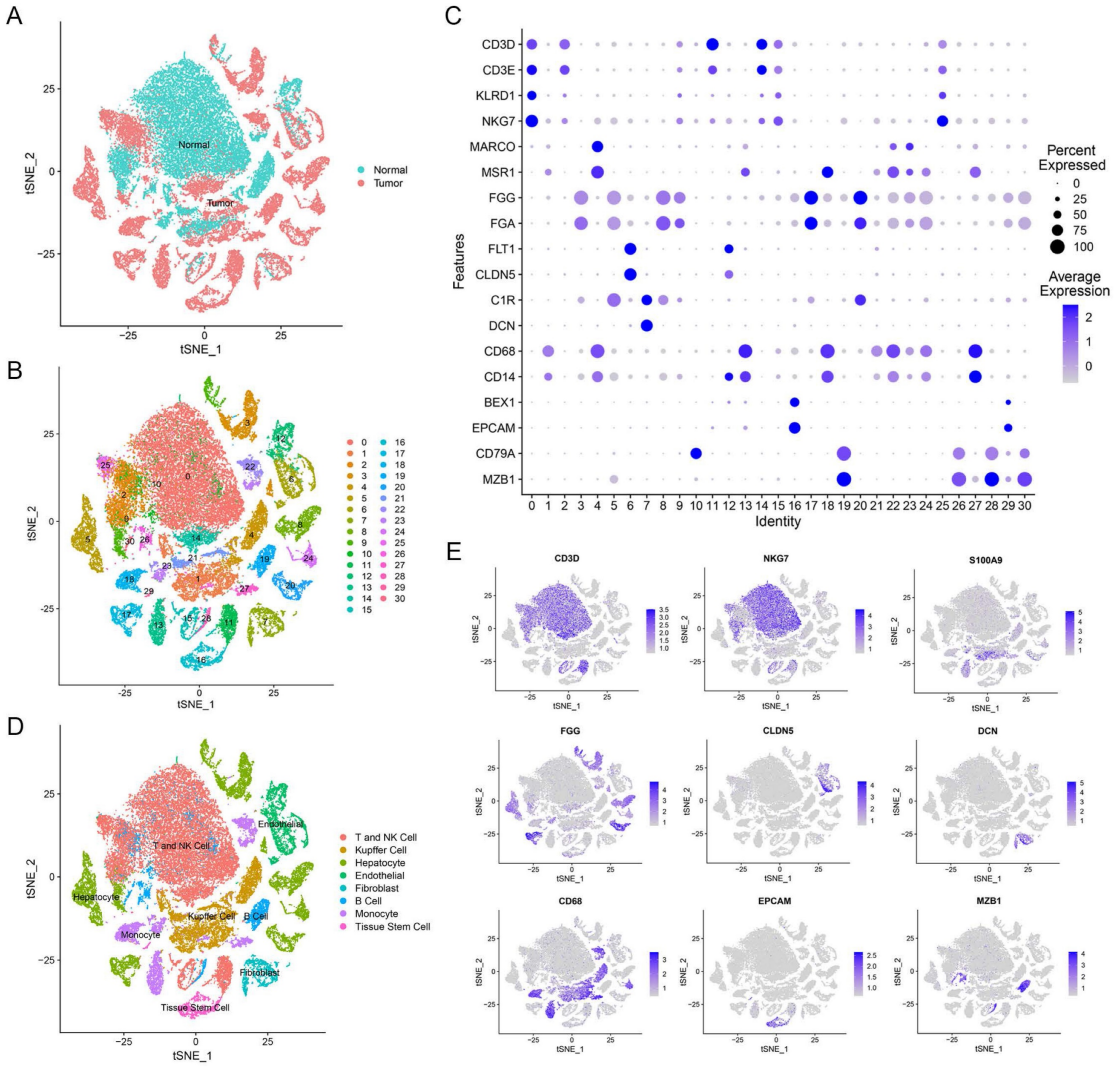
** Overview of scRNA-seq profiling from tumor and normal samples in HCC patients. (A)** Distribution of tissues in tSNE plot. **(B)** tSNE plot of 31 cell clusters.** (C)** Expression of major marker genes in 31 cell clusters. **(D)** Identification of cell types in tSNE plot. **(E)** Expression levels of marker genes in tSNE plot.

Our study utilized single-cell transcriptomic data from GSE149614, which included 10 primary tumor samples and 8 non-tumor samples from 10 HCC patients. In total, we analyzed 47,640 filtered cells and identified 31 distinct cell clusters through PCA and t-SNE dimensionality reduction (Figure 1A, B). These clusters were annotated with reference to Cell Marker 2.0 and the Human Protein Atlas (Figure 1C). By classifying T cells and NK cells into one cell type, we identified eight major cell types, including B cells, endothelial cells, fibroblasts, hepatocytes, T and NK cells, monocytes, tissue stem cells, and Kupffer cells (Figure 1D, E and Supplementary Figure 1). These subpopulations exhibited heterogeneity within HCC patient liver tissue and can provide a useful resource for exploring genes related to prognosis.

### Identification of NK Cell Marker Genes in Liver

**Figure 2:**
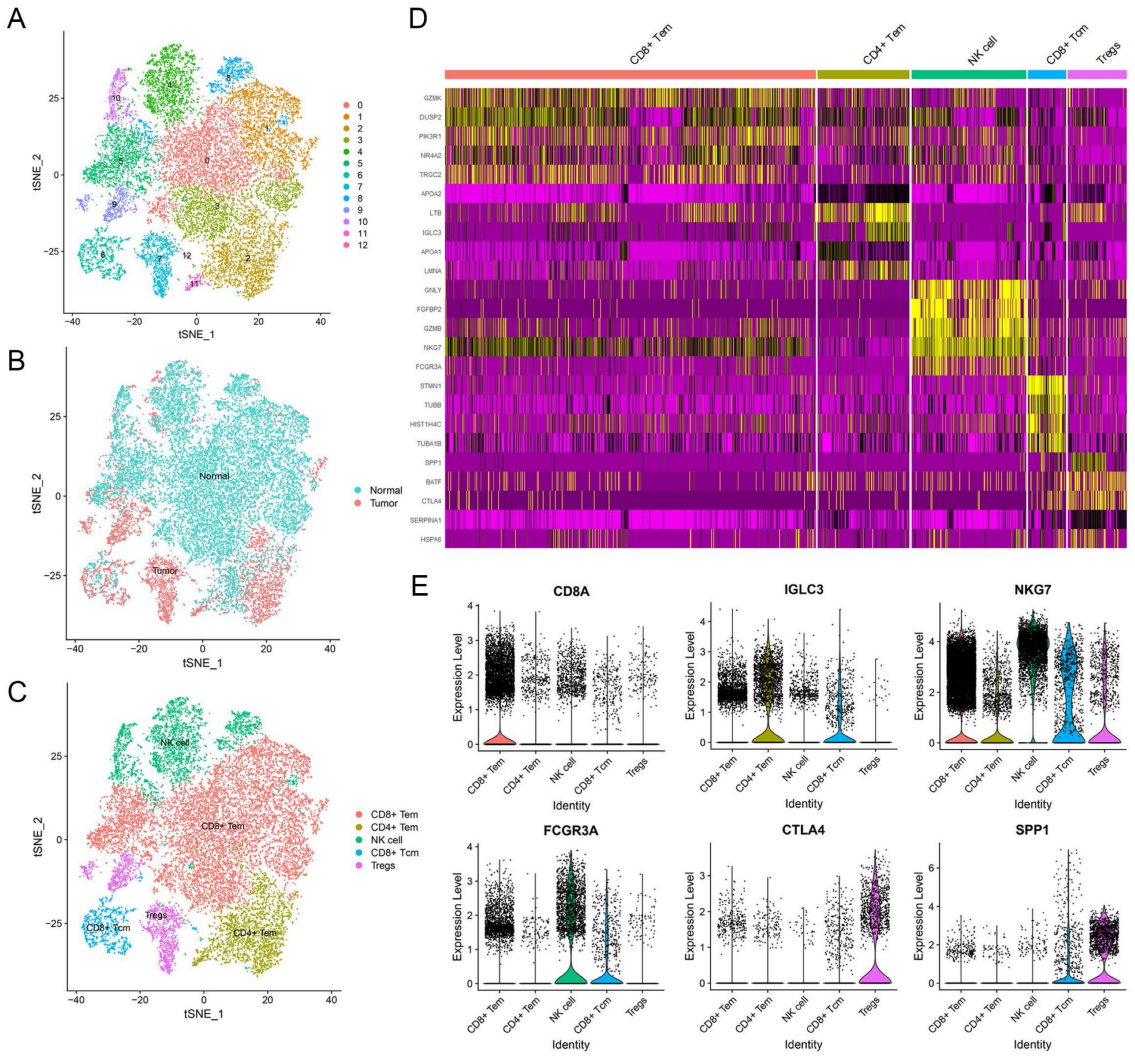
** Identification of NK cell marker genes by scRNA-seq analysis. (A)** tSNE plot of 13 subclusters in T and NK cell cluster.** (B)** Distribution of tissues in tSNE plot. **(C)** Identification of cell types in tSNE plot.** (D)** Heatmap showing the top five expression genes in each cell type. **(E)** Violin plots displaying characteristic gene expression in five subgroups.

To explore the unique genes expressed by NK cells, we partitioned the T and NK cell cluster consisting of 20,645 cells into 13 subclusters (Figure 2A). We identified five major cell types, which included NK cells, regulatory T cells (Treg cells), CD8^+^ central memory T cells (CD8^+^ Tcm cells), and CD4^+^ and CD8^+^ effector memory T cells (CD4^+^ and CD8^+^ Tem cells) (Figure 2C). In terms of their origin, Treg cells mainly originated from tumor tissues and displayed an immunosuppressive function (Figure 2B). Conversely, CD8^+^ Tem cells and NK cells were predominantly found in normal tissues (Figure 2B). CD4^+^ Tem cells and CD8^+^ Tcm cells, on the other hand, were derived from both normal and tumor tissues (Figure 2B). The subcluster of NK cells displayed distinct gene expression patterns (Figure 2D), with 79 genes being differentially expressed when compared to adaptive immune cells (Supplementary Table 1). Functional enrichment analysis revealed that the NK cell marker genes were mainly involved in NK cell mediated cytotoxicity, apoptosis, immune response, and cytolysis (Supplementary Figure 2).

### Construction of the Prognostic Model Based on NK Cell Marker Genes

To develop a prognostic signature based on the 79 NK cell marker genes, we utilized the TCGA-LIHC cohort, which includes 362 patients, as the training set and performed LASSO-COX dimensionality reduction analysis. We selected the nine genes that were most predictive and correlated, namely FABP5, GZMH, RAP1B, APOC1, PKM, EIF4A3, RGCC, IL7R and FTH1 to create the prognostic signature (Supplementary Figure 3). The risk score for each patient was calculated using the formula: Risk score = (0.051 × FABP5 expression) + (-0.022 × GZMH expression) + (0.071 × RAP1B expression) + (-0.004 × APOC1 expression) + (0.052 × PKM expression) + (0.129 × EIF4A3 expression) + (-0.055 × RGCC expression) + (-0.069 × IL7R expression) + (0.040 × FTH1 expression). Furthermore, we examined the relative gene expression in various clusters (Supplementary Figure 4), indicating that the nine marker genes have specific expression pattern.

**Figure 3:**
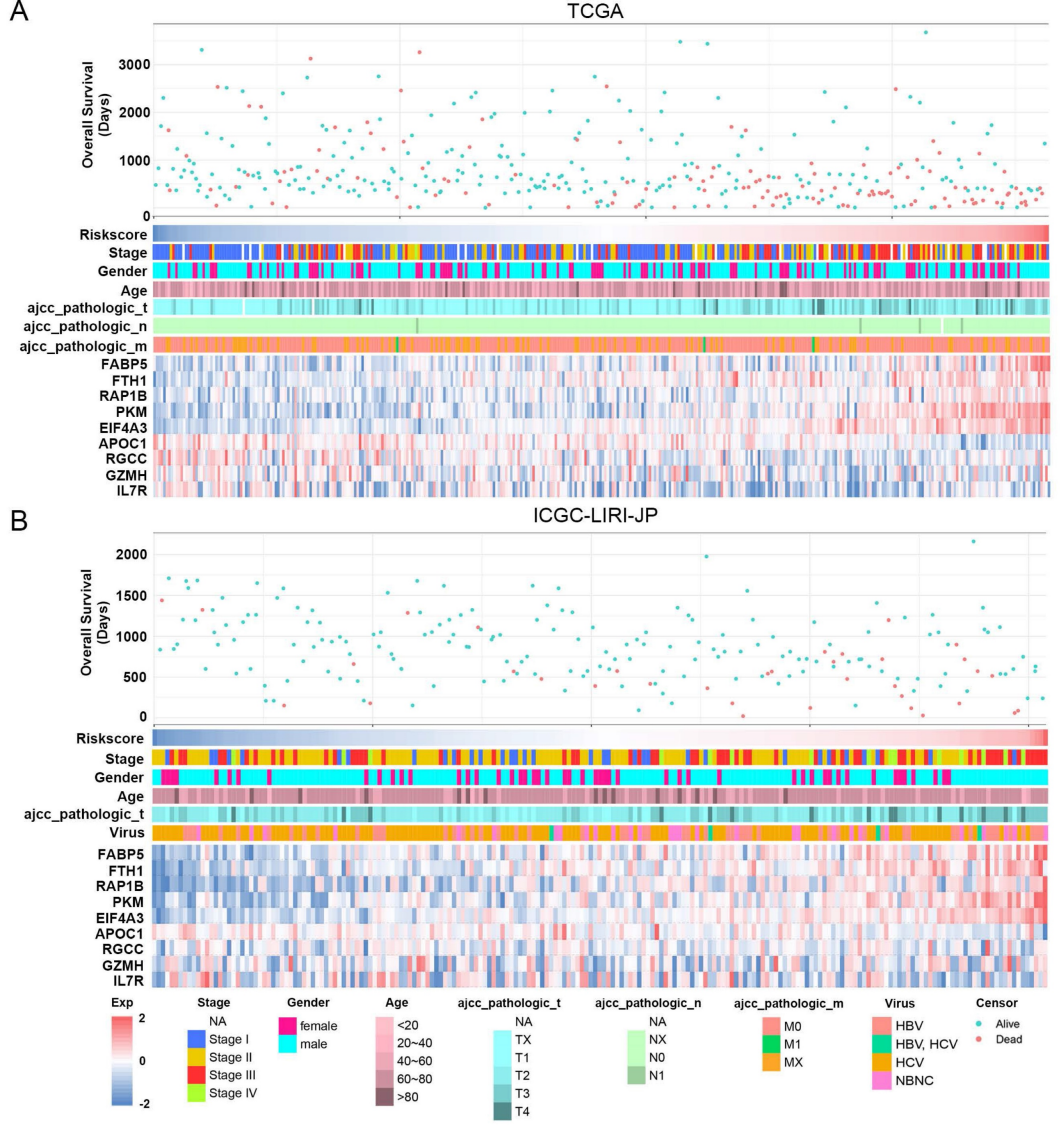
** Association between risk score and clinicopathological characteristics in HCC patients. (A)** Landscape of risk score related clinical-pathologic factors in the TCGA cohort. **(B)** Landscape of risk score related clinical-pathologic factors in the ICGC-LIRI-JP cohort. The point diagram shows the survival time and status of each HCC patient. The heatmap displays clinical-pathologic factors and expression levels of EABP5, FTH1, RAP1B, PKM, EIF4A3, APOC1, RGCC, GZMH, IL7R in ascending order of the risk score.

The study classified patients into low-risk (n = 181) and high-risk (n = 181) groups based on their median risk score of 0.956, which was calculated by arranging the risk score in ascending order. The patients in the high-risk group had a higher mortality rate and poor survival status as the risk score increased, as illustrated in Figure 3A. The heatmap demonstrates the expression level of NK cell marker genes, with five genes showing an increase in expression level as the risk score increased, whereas the expression level of others decreased. The high-risk group primarily comprised patients with advanced tumor stages, as shown in Figure 3A. Additional analysis revealed that there was a significant association between the risk score and tumor stage, while no significant correlation was found between the risk score and patient age or gender, as depicted in Figure 5A, B, and C.

**Figure 4:**
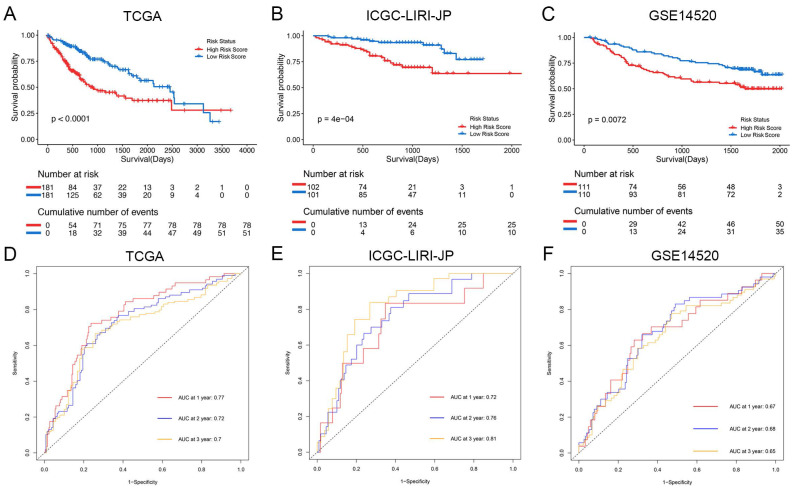
** Prognostic analysis of the NK cell related nine genes signature in three cohorts. (A-C)** Kaplan-Meier curves for overall survival in the TCGA cohort **(A)**, ICGC-LIRI-JP cohort **(B)**, and GSE14520 cohort **(C)**.** (D-F)** Time-dependent ROC curves show the predictive efficiency in the TCGA cohort **(D)**, ICGC-LIRI-JP cohort **(E)**, and GSE14520 cohort **(F)**.

The Kaplan-Meier analysis illustrated that patients in the high-risk group had significantly poorer overall survival rates compared to those in the low-risk group (Figure 4A). Additionally, to evaluate the predictive accuracy of the model, we calculated the area under the receiver operating characteristic curve for time-dependent overall survival. The AUC values were 0.77, 0.72, and 0.70 for 1, 2, and 3 years, respectively (Figure 4D), indicating a good predictive performance. Furthermore, the high-risk group consistently demonstrated lower overall survival rate than the low-risk group in different clinicopathological subgroups (Supplementary Figure 5).

### The NK Cell Gene Signature can Stably Predict the Prognosis of HCC Patients

**Figure 5:**
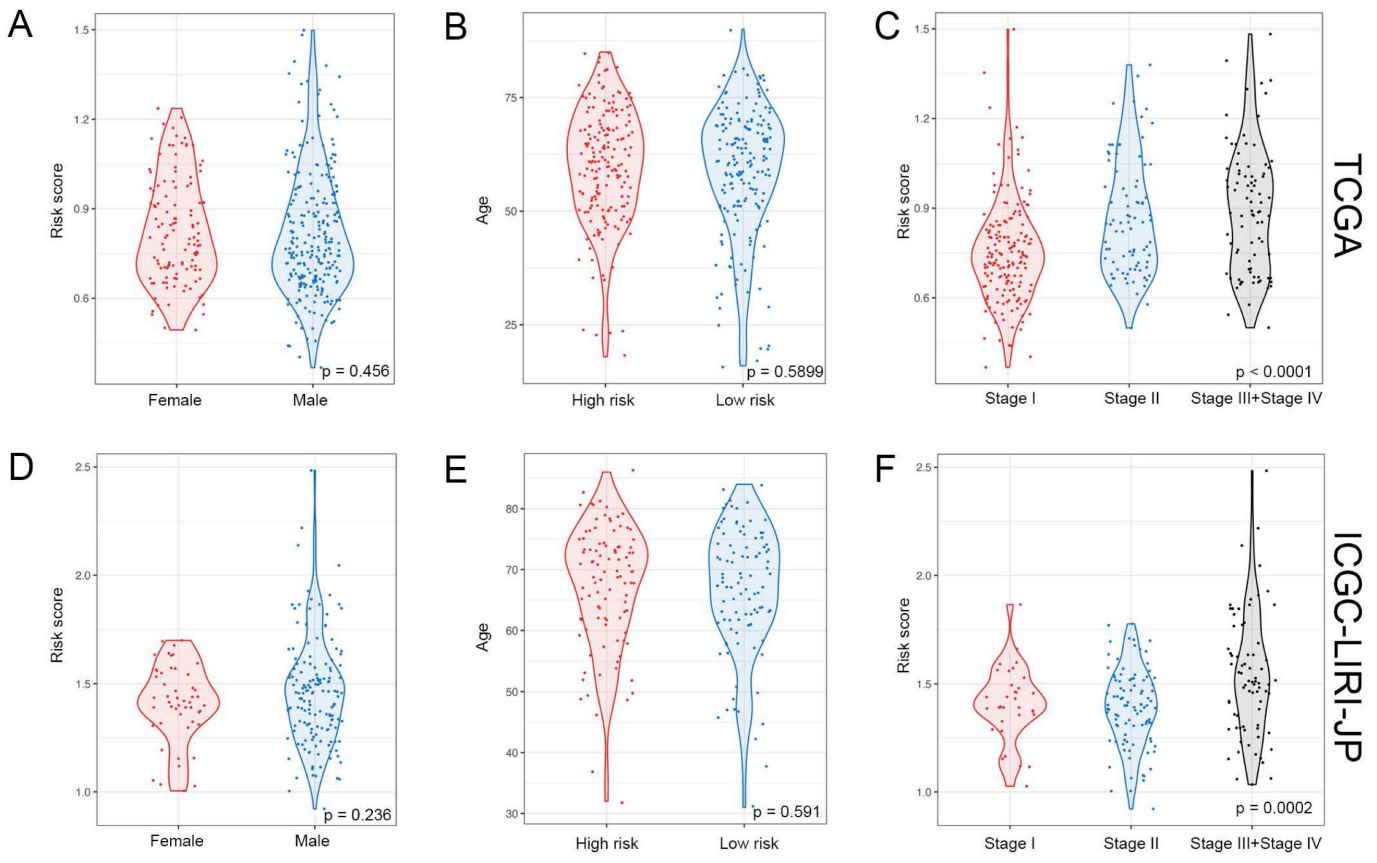
** Relationship between risk score and clinical-pathologic factors of HCC patients. (A-C)** Violin plots displaying the distribution of risk score across different clinical-pathologic factors in the TCGA cohort. **(D-F)** Violin plots displaying the distribution of risk score across different clinical-pathologic factors in the ICGC-LIRI-JP cohort. Student's *t*-test was used to verify the significance of differences between two groups, while One-way ANOVA was used to verify the significance of differences between three groups.

For validation purposes, we utilized two independent cohorts: the ICGC-LIRI-JP cohort and the GSE14520 cohort. Each patient in these cohorts was assigned a risk score based on the NK cell gene signature and then classified into either the low-risk or high-risk group based on the median risk score. In the ICGC-LIRI-JP cohort, patients with advanced tumor stage were observed more frequently in the high-risk group, and a higher number of deaths occurred in this group, indicating that the NK cell gene signature accurately predicts overall survival (Figure 3B). Patient age and gender did not differ significantly between the two risk groups, but there was an uneven distribution of viral infections across groups (Figure 3B). The expression level of 9 NK cell marker genes exhibited similar trends as the training set (Figure 3B). Further analysis demonstrated a significant association between risk score and tumor stage, but not with gender or age (Figure 5D, E, F). In both ICGC-LIRI-JP and GSE14520 cohorts, Kaplan-Meier analysis showed significantly lower overall survival rates for patients in the high-risk group (Figure 4B, C). The AUC values of 1, 2, and 3 years were 0.72, 0.76, and 0.81 in the ICGC-LIRI-JP cohort (Figure 4E) and 0.67, 0.68, and 0.65 in the GSE14520 cohort (Figure 4F), indicating good predictive accuracy of the model.

### Independent Prognostic Role of NK Cell Gene Signature for Patients With HCC

Univariate and multivariate COX analyses were performed to assess the efficacy of the risk score in comparison to other clinicopathologic characteristics. In the training set, both TNM stage (HR = 2.479, 95% CI = 1.706 - 3.602, *p*-value < 0.001) and the risk score (HR = 2.887, 95% CI = 1.949 - 4.275, *p*-value < 0.001) showed significant correlation with overall survival in the univariate COX regression analysis (Table 2). The multivariate Cox regression analysis showed that both TNM stage (HR = 2.156, 95% CI = 1.508 - 3.086, *p*-value < 0.001) and the risk score (HR = 2.572, 95% CI = 1.726 - 3.832, *p*-value < 0.001) were independent prognostic factors for the TCGA cohort (Table 2), confirming that the signature is an independent prognostic factor for HCC. The independence of the risk score in predicting prognosis was also validated in the ICGC-LIRI-JP cohort (Table 2) and GSE14520 cohort (Supplementary Table 2).

### Functional Enrichment Analysis of the NK Cell Gene Signature Related Genes

In order to investigate the potential mechanisms underlying the association between risk score and HCC prognosis, we conducted further analysis to explore the biological pathways and functions that are associated with the risk score. Initially, we identified 200 genes that showed significant correlation with the risk score through correlation analysis (Supplementary Table 3). We then performed GO and KEGG enrichment analyses on these genes. The GO analysis revealed that the risk score was primarily associated with biological processes such as cell division, mitotic spindle organization, mitotic cell cycle, and DNA replication (Supplementary Figure 6). Additionally, the KEGG analysis confirmed that these genes are closely involved in the cell cycle pathway (Supplementary Figure 6D).

### The Signature Associated with the TME

**Figure 6:**
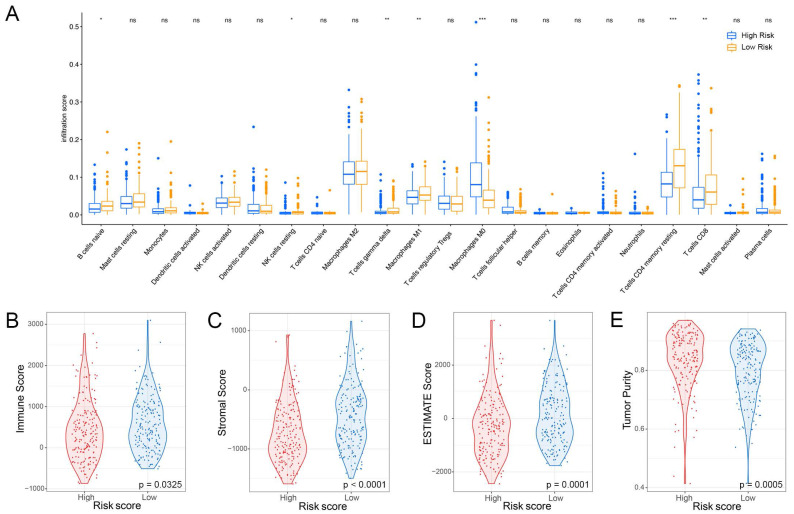
** Relationship between risk score and immune cell infiltration in the tumor microenvironment. (A)** Infiltration levels of 22 immune cell types in the TCGA cohort. *p < 0.05, **p < 0.01, ***p < 0.001 determined by Wilcoxon test. **(B)** Differences in immune score,** (C)** stromal score, **(D)** ESTIMATE score, and **(E)** tumor purity between high-risk and low-risk groups; *p*-values determined by Student`s *t*-test.

The liver has a crucial role in the innate immune response, with a significantly higher percentage of natural killer (NK) cells present compared to the spleen or peripheral blood 13. In this study, we aimed to investigate the association between the NK cell marker gene signature and the tumor microenvironment (TME). To assess the level of immune cell infiltration, we utilized the CIBERSORT algorithm. Our findings suggest that patients at high-risk group have a higher proportion of M0 macrophages but a lower proportion of B cells naïve, resting NK cells, T cells gamma delta, M1 macrophages, resting T cells CD4 memory, and CD8^+^ T cells (Figure 6A). Additionally, we performed a correlation analysis between the signature and immune cell infiltration and observed a positive correlation between the risk score and neutrophils, eosinophils, and macrophages, whereas T cells, NK cells, and mast cells exhibited a negative correlation (Supplementary Figure 7). By employing the ESTIMATE algorithm, we found that patients at high-risk group had lower immune score, stromal score, ESTIMATE score, and higher tumor purity compared to patients at low-risk group (Figure 6B-E).

### The Individualized Prediction Model Showed Robust Predictive Accuracy

**Figure 7:**
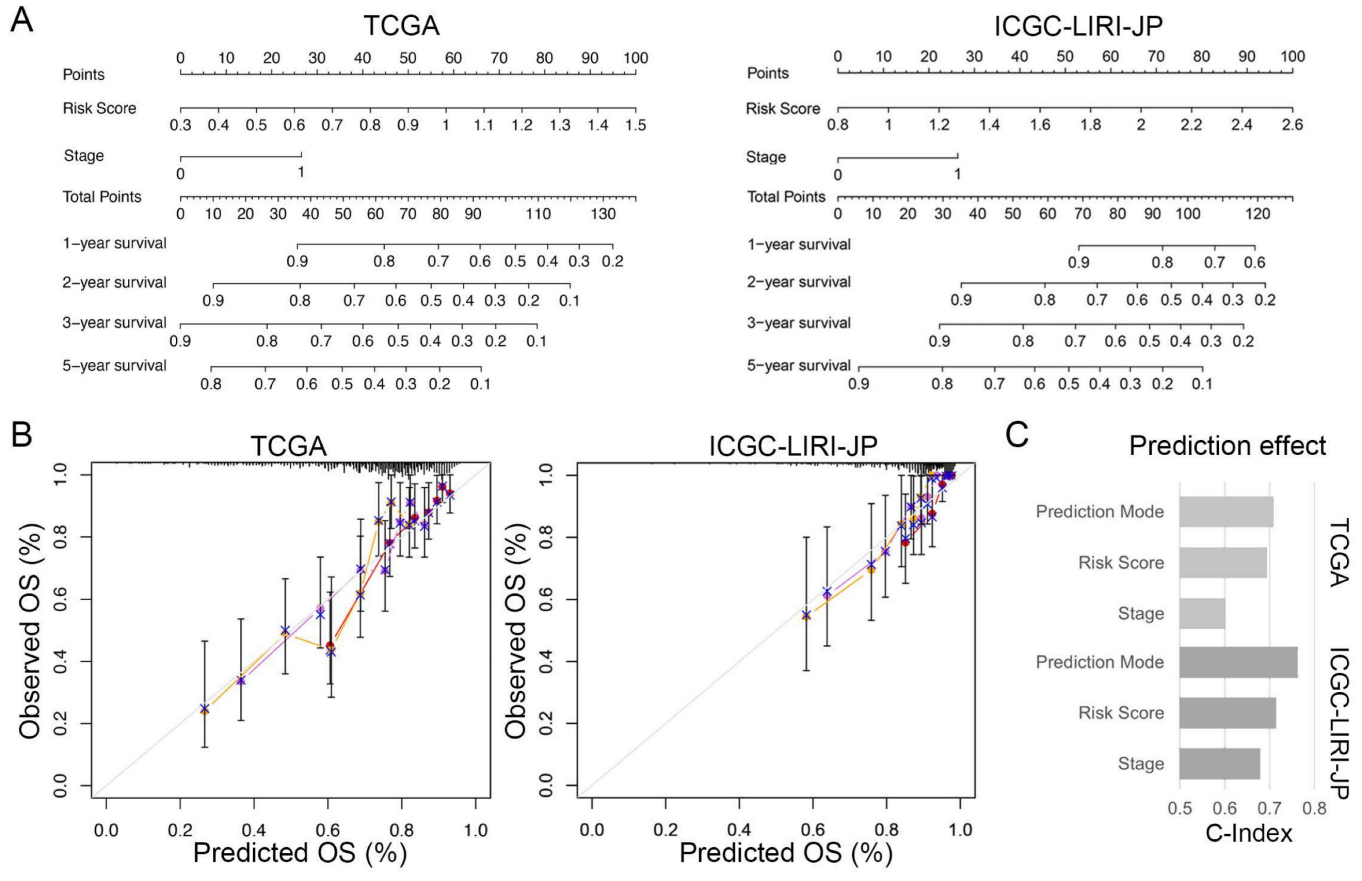
** Individualized prediction models for overall survival in HCC. (A)** Nomogram predicting 1-, 2-, 3-, and 5-year OS of HCC patients based on the TCGA and ICGC-LIRI-JP cohorts. **(B)** Predictive accuracy assessment of TCGA and ICGC-LIRI-JP cohorts. **(C)** Evaluated of the predictive effect of the individualized prediction model on OS in HCC patients using the C-Index.

To enhance the practicality of the prognostic signature, we created a personalized prediction model based on the independent prognostic factors. We meticulously chose several variables, including age, gender, grade, tumor stage, family history, vascular invasion, and risk score, to construct the nomogram. These variables were selected due to their clinical relevance and potential correlation with prognosis. We performed both univariate and multivariate Cox regression analyses to determine the independent prognostic factors among these variables (Table 2). Our final model incorporated the risk score and TNM staging as crucial prognostic factors for both the training and validation sets (Figure 7A). Notably, the total score derived from the nomogram accurately predicted the survival rate of HCC patients. To evaluate the performance of the personalized prediction model, we compared the predicted and actual clinical survival outcomes in these sets (Figure 7B). The C-index values were calculated as 0.702 and 0.762 in the training and validation sets, respectively, indicating good discrimination and predictive accuracy of our model (Figure 7C). Additionally, we observed that patients with advanced TNM stage and higher risk scores exhibited significantly worse prognosis. These findings highlight the clinical utility of our prognostic model in stratifying HCC patients based on their risk score and TNM staging, providing valuable information for prognostic assessment and treatment decision-making.

**Figure 8:**
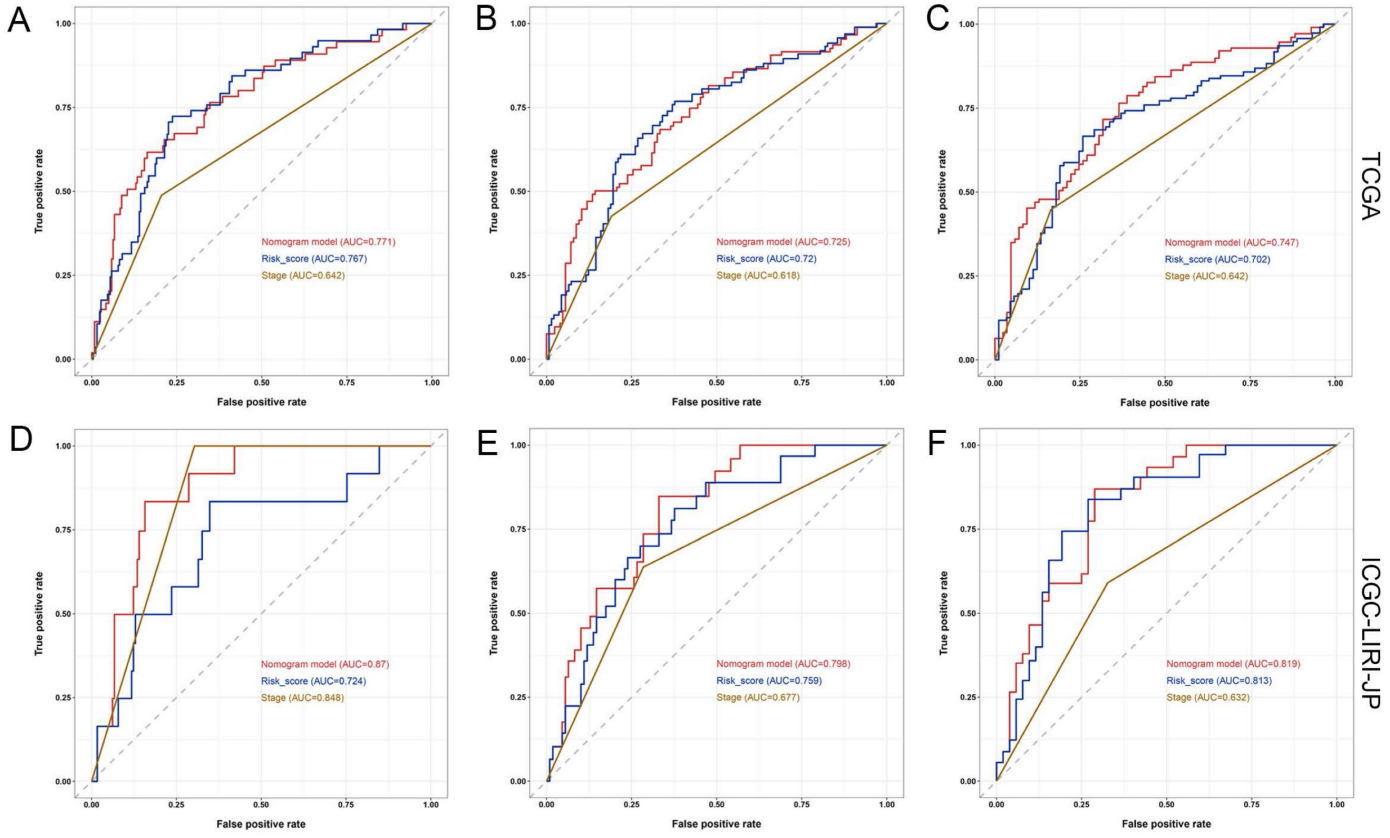
** Comparison of the predictive performance between nomogram, risk score, and TNM stage.** (A-C) ROC curve and AUCs for 1-year (A), 2-year (B), and 3-year (C) overall survival (OS) of HCC patients based on the TCGA cohort. (D-F) ROC curve and AUCs for 1-year (D), 2-year (E), and 3-year (F) OS of HCC patients based on the ICGC-LIRI-JP cohort.

Furthermore, the ROC analysis depicted in Figure 8 highlights the superior predictive performance of the nomogram compared to both tumor stage and risk score in forecasting the 1-year, 2-year, and 3-year prognosis within the training and validation sets. Within the training set, the nomogram achieved impressive AUC values of 0.771, 0.725, and 0.747 for the 1-year, 2-year, and 3-year prognoses, respectively (Figure 8A-C). In contrast, the tumor stage demonstrated relatively lower AUC values of 0.642, 0.618, and 0.642 for the corresponding time periods (Figure 8A-C). Similarly, in the validation set, the nomogram exhibited strong predictive capabilities with AUC values of 0.87, 0.798, and 0.819, surpassing the AUC values of 0.848, 0.677, and 0.632 attained by the tumor stage (Figure 8D-F). These compelling findings suggest that the nomogram holds significant potential as a more precise prognostic indicator for outcomes in the HCC patients.

## Discussion

Single-cell RNA sequencing (scRNA-seq) has become a powerful tool in oncology for analyzing complex cell populations and developing clinical diagnostic markers. In hepatocellular carcinoma (HCC), the immune cell landscape in both intrahepatic and tumor tissues has been studied to gain insights into disease progression. Specifically, decreased NK cell function has been linked to cancer progression and poor prognosis 13. To identify potential diagnostic markers from NK cells, we analyzed scRNA-seq dataset (GSE149614) and identified 79 candidate NK cell marker genes. Using LASSO-COX regression, we identified a set of critical genes associated with the prognosis of HCC patients and developed a nine-gene risk model with a high prediction effect. Our findings suggest that this model may be a valuable biomarker for predicting the efficacy of immunotherapeutic responses in cancer patients. Other studies have also utilized scRNA-seq to examine macrophage marker genes in breast cancer 28 and NK cell marker genes in lung adenocarcinoma 25. Overall, scRNA-seq technology enables comprehensive molecular profiling of innate immune cells and can aid in the identification of potential prognostic biomarkers.

The gene signature utilized in this study comprised of nine NK cell marker genes, namely FABP5, GZMH, RAP1B, APOC1, PKM, EIF4A3, RGCC, IL7R, and FTH1. Most of these genes are linked to the immune cell function or prognosis of hepatocellular carcinoma. Fatty acid binding protein 5 (FABP5) is a member of the FABP family that is responsible for the transportation and binding of fatty acids into cells. FABP5 has been associated with the development and onset of inflammation, metabolic disorders, and several types of tumors, as reported in previous studies 35, 36. In triple negative breast cancer (TNBC), FABP5 has been found to promote metastasis by inhibiting EGFR proteasome degradation 37. FABP5 has also been reported to enhance lipid accumulation and cell proliferation by boosting Hypoxia-inducible factor-1 alpha (HIF-1α) activity in HCC 38. Moreover, FABP5 promotes tumor angiogenesis and activates the IL6/STAT3/VEGFA pathway in HCC, as evidenced by studies 39. Within the tumor microenvironment of HCC, FABP5 fosters lipid accumulation in monocytes/macrophages, modifies programmed death-ligand 1 (PD-L1) expression in regulatory T cells, and promotes immune tolerance 40. In addition, FABP5 has been identified as a crucial prognostic factor in various tumors, including lung adenocarcinoma (LUAD), clear cell renal cell carcinoma (ccRCC), and multiple myeloma (MM), among others 41, 42. Apolipoprotein C1 (APOC1) is a member of the apolipoprotein family that participates in lipoprotein metabolism and cancer development 43. In clear cell renal carcinoma, APOC1 facilitates the activation of signal transducer and activator of transcription (STAT3) and promotes the metastasis of tumor cells 44. In colorectal cancer, APOC1 modulates cell proliferation and motility via the mitogen-activated protein kinase (MAPK) pathway 45. APOC1 has been suggested as a promising biomarker for the diagnosis and prognosis of various cancers, such as ovarian cancer and papillary thyroid carcinoma 46, 47.

Eukaryotic initiation factor 4A-3 (EIF4A3) is a DEAD box helicase that functions as a core constituent of the exon junction complex (EJC). It is involved in RNA metabolic processes and has been found to play a significant role in tumor progression 48. In gastric cancer, EIF4A3 binds to circRNA_0074027, promoting the proliferation and migratory capacity of tumor cells 49. Moreover, EIF4A3 has been implicated in the biogenesis of circTOLLIP, a circular RNA derived from exons of Toll-interacting protein (TOLLIP), which activates the PBX3/EMT signaling pathway, promoting HCC cell proliferation and metastasis 50. Additionally, EIF4A3 has been demonstrated to regulate the splicing of FGFR4, which influences HCC aggressiveness and patient survival 51. FTH1, a critical factor in cancer progression, is an iron regulatory protein that inhibits ferroptosis by binding Fe^2+^ 52, 53. Recent studies have revealed that FTH1 upregulation is associated with metastasis and poor prognosis of head and neck squamous cell carcinoma (HNSCC) patients 54, 55, and FTH1 has also been used as a prognostic marker in HCC 56.

Furthermore, GZMH, a highly conserved serine protease, is constitutively expressed at high levels in NK cells and is a crucial component of NK cell-mediated cytolysis 57, 58. By targeting both mitochondrial and nuclear components, GZMH induces cell death, expanding the range of cell death-inducing mechanisms in the innate immune system 59. The interleukin-7 receptor (IL-7R), composed of a high-affinity α-receptor (IL7R or CD127) and a common γ-chain (CD132) shared with other cytokine receptors, is a heterodimeric receptor 60. IL7R has been shown to be expressed on NK cells and activate IL-7 signaling, which leads to CD56^bright^ NK cell activation, IFN-γ release, and NK cell lytic activity 61, 62. IL7R has also been identified as a potential biomarker for HCC, reflecting the tumor microenvironment and prognosis 63, 64.

Pyruvate kinases M (PKM) are critical rate-limiting enzymes in glucose metabolism, consisting of PKM1 and PKM2 isoforms 65. PKM1 primarily generates energy in brain and muscle tissues under hypoxic conditions, whereas PKM2 is predominantly expressed in cells with unrestricted proliferation potential, and its activation stimulates tumorigenesis and development through the Warburg effect 66, 67. High expression of PKM2 has been linked to poor prognosis in acute leukemia and intrahepatic cholangiocarcinoma 68, 69.

Rap1b is a ubiquitous GTP-binding protein that plays a vital role in modulating various cellular functions, including cell proliferation, migration, polarity, and endothelial cell adhesion 70, 71. In HCC, Rap1b promotes the proliferation and migration of tumor cells by enhancing the expression of Twist1 72. In several types of tumors, such as colorectal cancer, renal cell carcinoma, and melanoma, reducing the expression of RAP1B effectively inhibits tumor cell proliferation and migration 73-75. RGCC, also known as RGC-32 (response gene to complement 32), is a complement response gene that plays a critical role in regulating the cell cycle, immune response, and tumor metastasis 76, 77. Overexpression of RGCC promotes cell proliferation and invasion and is associated with poor prognosis in patients with colon adenocarcinoma (COAD) 78. In lung adenocarcinoma (LUAD), RGCC stimulates epithelial-mesenchymal transition (EMT), enhances cell migration and invasion, and reduces MMP-2 and MMP-9 protein activity and expression 79. These genes identified in the NK cell marker gene signature could serve as potential targets for laboratory-based experimental design to elucidate the molecular mechanisms underlying HCC. Identification of these targets may facilitate the discovery of novel therapeutic interventions that can improve patient outcomes.

Although the results of this study show promise, it is important to acknowledge several limitations. Firstly, the small sample size and limited availability of comprehensive clinical information may have influenced the accuracy and robustness of the predictive model. To improve its reliability and applicability in clinical settings, it is crucial to validate and refine the model using larger cohorts with more extensive clinical data. Secondly, focusing solely on NK cell marker genes in the prognostic signature may restrict its predictive effectiveness due to the heterogeneous nature of the tumor microenvironment. Exploring the potential contribution of other immune cell types or molecular markers is necessary to enhance the predictive effectiveness of the signature, considering the complex interactions within the tumor microenvironment. Additionally, while the current study primarily focused on gene expression levels and their association with prognosis, investigating the relationship between protein levels of the signature genes and patient outcomes in liver cancer would provide a more comprehensive understanding of the underlying mechanisms. Future research could incorporate quantitative proteomics techniques to optimize the predictive capacity of the signature by elucidating protein-level alterations and their impact on prognosis.

The development of this prognostic signature has important implications for personalized treatment and clinical decision-making in hepatocellular carcinoma. Accurately assessing the prognostic risk of patients can enable clinicians to identify individuals who are more likely to benefit from specific therapeutic interventions or require intensified follow-up. Furthermore, the identification and functional annotations of novel NK cell marker genes contribute to our understanding of the role of NK cells in liver cancer and open doors for targeted immunotherapies.

## Conclusion

In essence, this study utilized RNA-seq and scRNA-seq methodologies to investigate the expression of NK cell marker genes. Subsequently, a prognostic model consisting of nine genes was developed. The model displayed strong predictive capabilities in determining the survival prognosis of individuals with HCC, thereby indicating its potential as a personalized prognostic biomarker that can assist in clinical decision-making. This biomarker holds particular value in identifying eligible patients who could potentially gain advantages from immunotherapy.

## Supplementary Material

Supplementary figures and tables.

## Figures and Tables

**Table 1:** Clinical characteristics of the HCC patients used in this study.

	TCGA	ICGC-LIRI-JP	GSE14520
**No. of patients**	362	203	221
**Age (median, range)**	61 (16-90)	69 (31-89)	50 (21-77)
**Gender (%)**			
Female	118 (32.6%)	50 (24.6%)	30 (13.6%)
Male	244 (67.4%)	153 (75.4%)	191 (86.4%)
**Family History (%)**			
No	203 (56.1%)	NA	NA
Yes	110 (30.4%)	NA	NA
unknown	49 (13.5%)	NA	NA
**Stage (%)**			
Stage Ⅰ	169 (46.7%)	33 (16.3%)	93 (42.1%)
Stage Ⅱ	84 (23.2%)	96 (47.3%)	77 (34.8%)
Stage Ⅲ	81 (22.4%)	59 (29.1%)	49 (22.2%)
Stage Ⅳ	4 (1.1%)	15 (7.4%)	0 (0.0%)
Unknown	24 (6.6%)	0 (0.0%)	2 (0.9%)
**AFP (ng/ml)**			
≤300	211 (58.3%)	NA	118 (53.4%)
>300	64 (17.7%)	NA	100 (45.2%)
Unknown	87 (24.0%)	NA	3 (1.4%)
**Grade (%)**			
Grade 1	55 (15.2%)	NA	NA
Grade 2	175 (48.3%)	NA	NA
Grade 3	115 (31.8%)	NA	NA
Grade 4	12 (3.3%)	NA	NA
Unknown	5 (1.4%)	NA	NA
**Vascular Invasion (%)**			
None	204 (56.4%)	NA	NA
Yes	104 (28.7%)	NA	NA
Unknown	54 (14.9%)	NA	NA

**Table 2:** Univariable and multivariable Cox regression analysis of the signature characteristics in the TCGA and ICGC-LIRI-JP cohorts.

Characteristics	Univariable analysis			Multivariable analysis		
	HR	95% CI	P-Value	HR	95% CI	P-Value
**TGCA**						
**Age**						
≤60	1.0 (ref)					
>60	1.252	0.882-1.776	0.209			
**Gender**						
Female	1.0 (ref)					
Male	0.825	0.578-1.178	0.290			
**Grade**						
G1+G2	1.0 (ref)					
G3+G4	1.122	0.780-1.612	0.535			
**Family History**						
No	1.0 (ref)					
Yes	1.183	0.818-1.712	0.372			
**Vascular Invasion**						
No	1.0 (ref)					
Yes	1.346	0.885-2.046	0.165			
**TNM stage**						
I + II	1.0 (ref)			1.0 (ref)		
III + IV	2.479	1.706-3.602	<0.001	2.156	1.508-3.086	<0.001
**Risk score**						
Low	1.0 (ref)			1.0 (ref)		
High	2.887	1.949-4.275	<0.001	2.572	1.726-3.832	<0.001
**ICGC-LIRI-JP**						
**Age**						
≤60	1.0 (ref)					
>60	0.976	0.426-2.237	0.955			
**Gender**						
**Female**	1.0 (ref)					
Male	0.536	0.265-1.084	0.083			
**TNM stage**						
I + II	1.0 (ref)			1.0 (ref)		
III + IV	2.828	1.445-5.535	0.002	2.424	1.233-4.767	0.01
**Virus**						
No	1.0 (ref)					
Yes	1.655	0.506-5.413	0.405			
**Risk score**						
Low	1.0 (ref)			1.0 (ref)		
High	3.565	1.686-7.537	0.001	3.176	1.485-6.793	0.003

HR, hazard ratio; CI, confidence interval; ref, reference category.
