# Supplementary material for: A Novel Prognostic Signature of comprising Nine NK Cell signatures Based on Both Bulk RNA Sequencing and Single-Cell RNA Sequencing for Hepatocellular Carcinoma
Source: J Cancer. 2023 Jul 16;14(12):2209–23. doi: 10.7150/jca.85873 (PMC10414035; doi:10.7150/jca.85873)
Supplement: Supplementary file 1 — Supplementary figures and tables. [file jcav14p2209s1.pdf]

Supplementary Materials

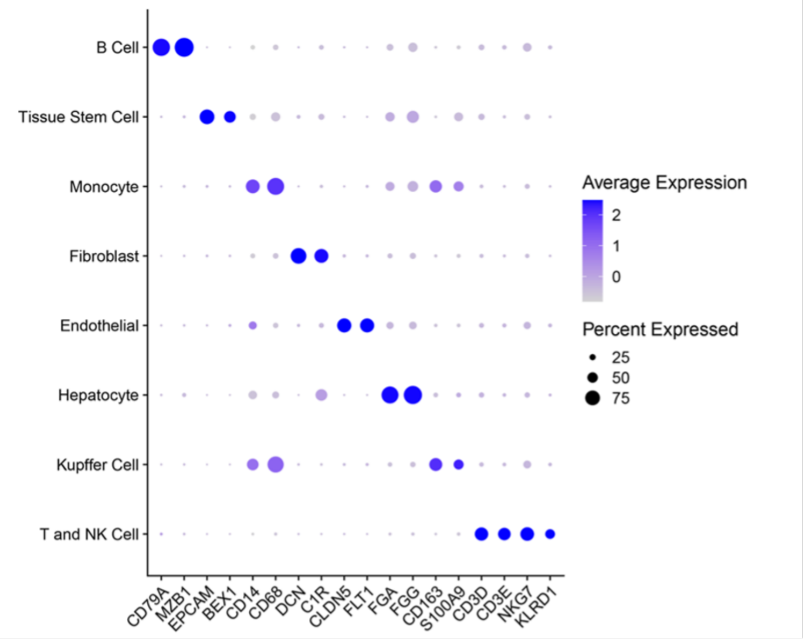

Supplementary Figure 1. Bubble plot showing the expression of cell marker genes in each cell types. Bubble intensity of color indicates the average expression in a particular cluster and bubble size represents the percent of cells expressing the gene in that cluster.

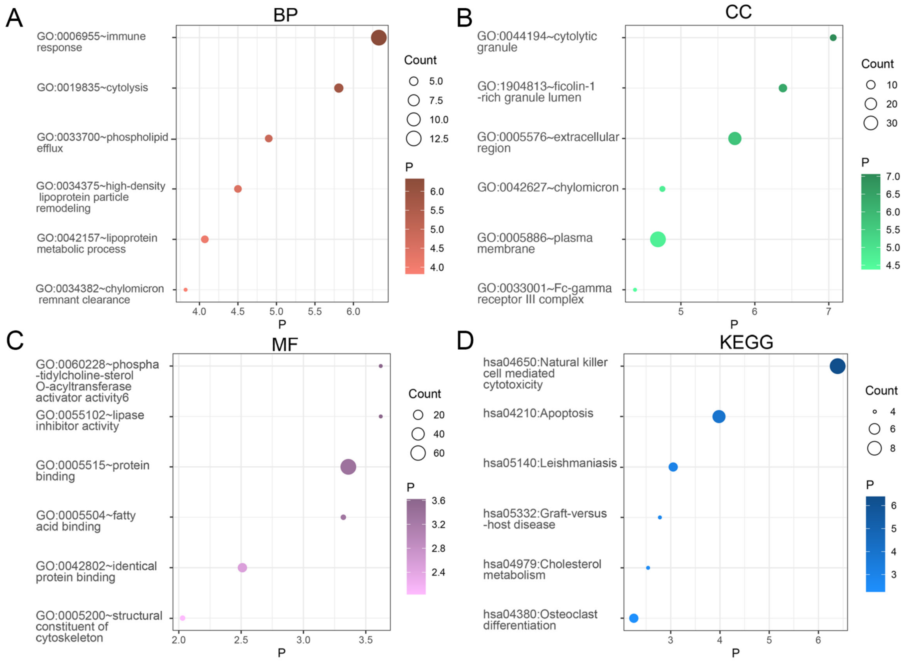

Supplementary Figure 2. Functional enrichment analysis of NK cell marker genes of LIHC. (A) Biological processes (BP), **(B)** cellular components (CC), **(C)** molecular

functions (MF) and **(D)** KEGG pathway.

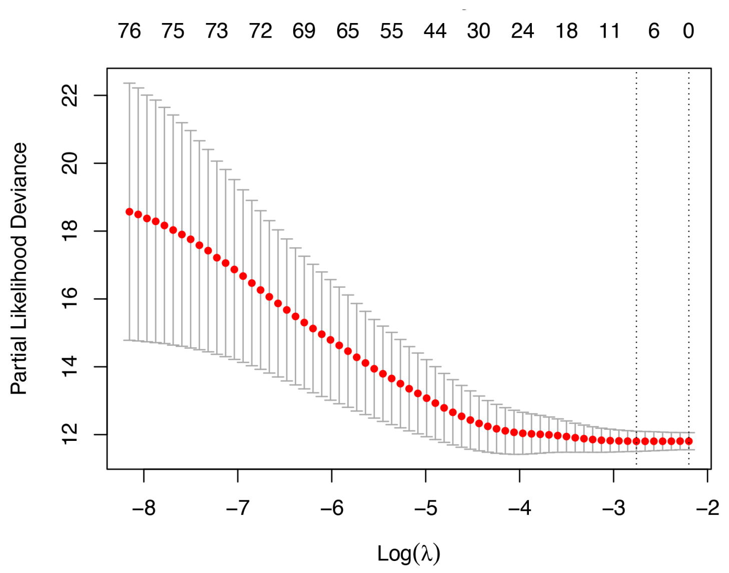

Supplementary Figure 3. LASSO-COX regression identified nine NK cell marker gene signature.

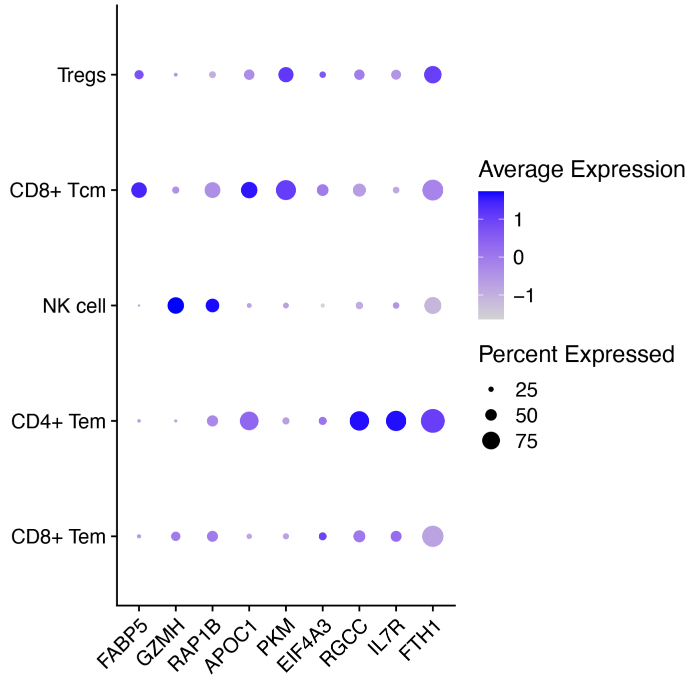

Supplementary Figure 4. Bubble plot showing the expression of NK cell marker genes in different cell types.

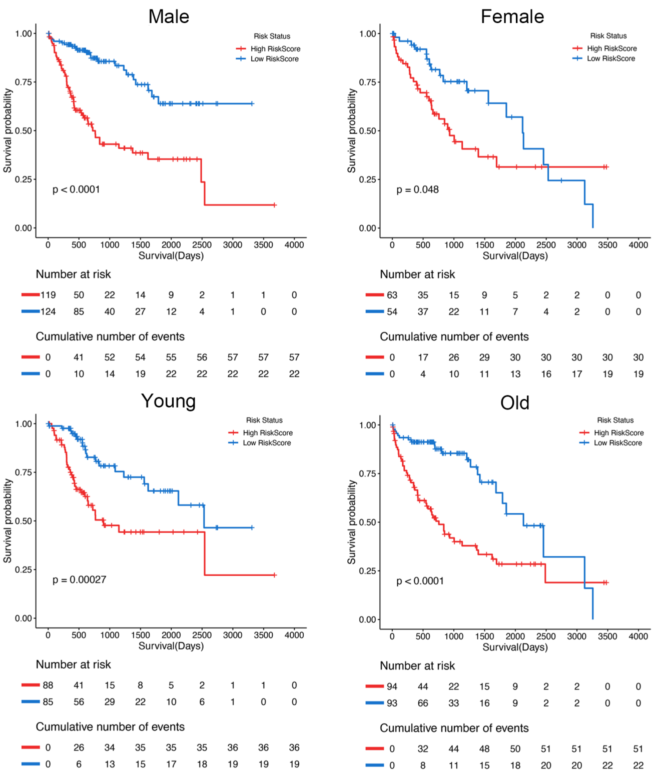

Supplementary Figure 5. Verification of the prognostic predictive capability of NK cell signature in different clinical subgroups. Kaplan-Meier curves of OS in male, female, young and old patients based on risk score in TCGA cohort.

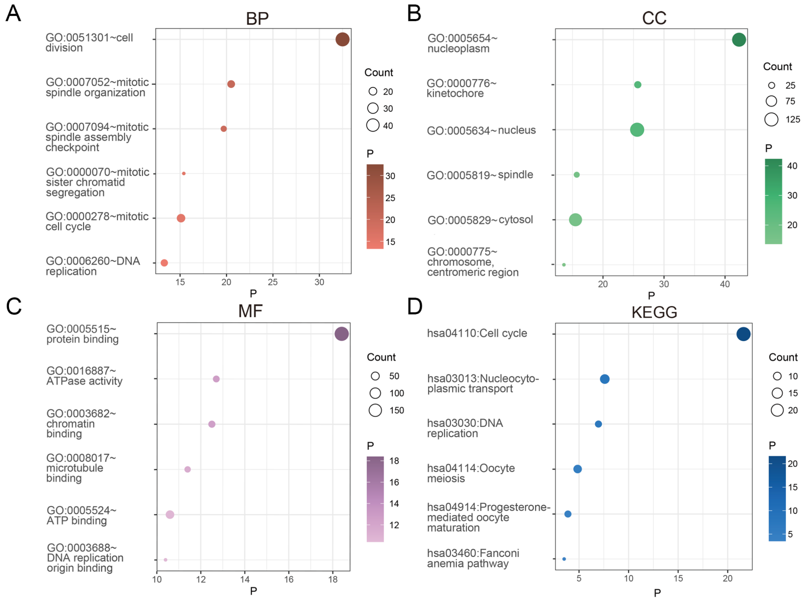

Supplementary Figure 6. Functional enrichment analysis of the risk score related genes in TCGA cohort. (A) Biological processes (BP), **(B)** cellular components (CC), **(C)** molecular functions (MF) and **(D)** KEGG pathway.

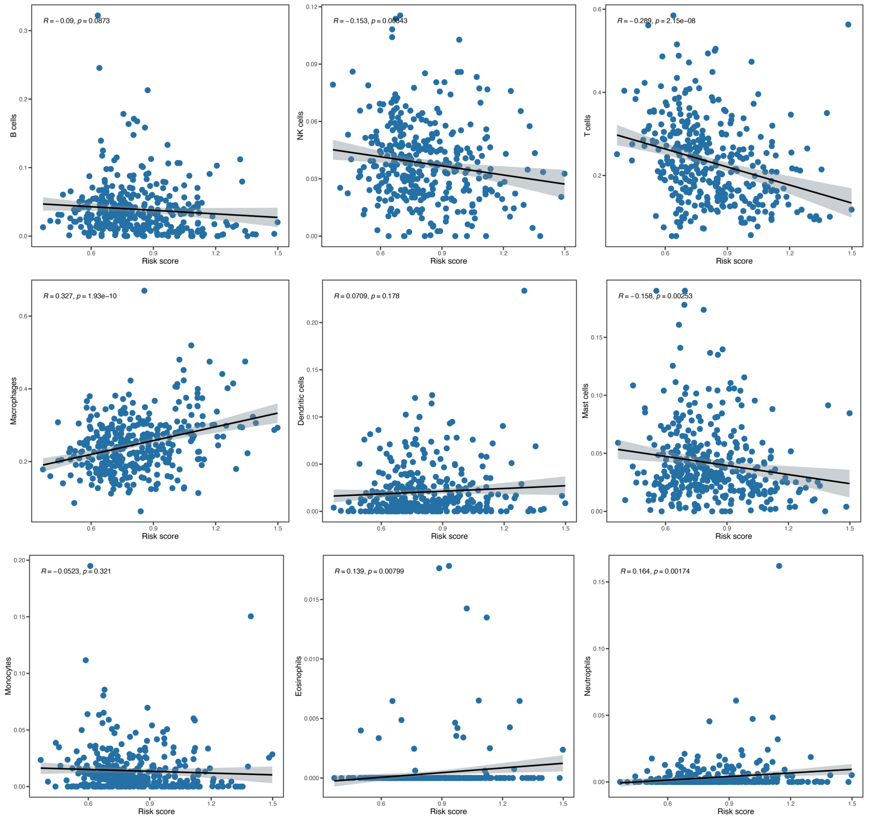

Supplementary Figure 7. The correlation analysis between estimated immune cell infiltration level and risk score.

Supplementary Table 1. 79 NK cell marker genes.

No.	Gene	No.	Gene	No.	Gene	No.	Gene
1	FCGR3A	21	IFITM2	41	S1PR5	61	AKNA
2	GNLY	22	TRAC	42	MYO1F	62	MIF
3	FGFBP2	23	GPR183	43	APOE	63	JUN
4	CTSW	24	COTL1	44	ZEB2	64	GSTP1
5	PRF1	25	RGS1	45	CXCR6	65	CDC42SE1
6	KLRD1	26	ITGB2	46	HOPX	66	ALB
7	GZMH	27	TRAT1	47	IL32	67	CLIC3
8	GZMB	28	RGCC	48	RAP1B	68	PYHIN1
9	NKG7	29	GZMK	49	AREG	69	FABP5
10	SPON2	30	ZFP36L2	50	PLP2	70	APOC3
11	KLRF1	31	BTG1	51	EFHD2	71	MAPK1
12	PLAC8	32	LITAF	52	NEAT1	72	LMNA
13	CST7	33	FCER1G	53	HMGN1	73	DSTN
14	TYROBP	34	IL7R	54	PPP1R2	74	IFITM3
15	LTB	35	SARAF	55	CMC1	75	HSPB1
16	IFITM1	36	APOA2	56	GZMM	76	HLA-DRA
17	CD247	37	FTH1	57	SERPINA1	77	PKM
18	RPL13	38	BIN2	58	CCND3	78	TUBA1B
19	JUNB	39	VIM	59	APOC1	79	TUBB
20	RPS2	40	DUSP1	60	EIF4A3		

Supplementary Table 2. Univariable and multivariable Cox regression analysis of the GSE14520 cohort.

Characteristics	Univariable analysis			Multivariable analysis		
	HR	95% CI	P-Value	HR	95% CI	P-Value
			GSE14520			
**Age**						
≤60	1.0 (ref)					
>60	0.805	0.453-1.429	0.458			
**Gender**						
Female	1.0 (ref)					
Male	1.658	0.800-3.435	0.174			
**AFP**						
≤300ng/ml	1.0 (ref)			1.0 (ref)		
>300ng/ml	1.655	1.077-2.542	0.021	1.215	0.770-1.917	0.403
**Main Tumor Size**						
Small	1.0 (ref)			1.0 (ref)		
Large	2.085	1.353-3.213	0.001	1.122	0.644-1.955	0.685
**Cirrhosis**						
No	1.0 (ref)			1.0 (ref)		
Yes	4.756	1.169-19.346	0.029	4.084	0.997-16.739	0.051
**TNM stage**						
I + II	1.0 (ref)			1.0 (ref)		
III	3.41	2.175-5.349	<0.001	2.643	1.489-4.691	0.001
**Risk score**						
Low	1.0 (ref)			1.0 (ref)		
High	1.879	1.214-2.909	0.005	1.612	1.024-2.538	0.039

HR, hazard ratio; CI, confidence interval; ref, reference category.

Supplementary Table 3. 200 genes that closely correlated with the signature.

No.	Gene	R	p value	No.	Gene	R	p value
1	KPNA2	0.778	9.24E-75	41	CCT6A	0.706	7.72E-56
2	TRIP13	0.771	1.28E-72	42	KIF20A	0.704	1.50E-55
3	KIF2C	0.756	2.49E-68	43	RAD51	0.703	2.76E-55
4	EFTUD2	0.755	5.73E-68	44	NCAPG	0.703	3.47E-55
5	PRR11	0.753	1.87E-67	45	CHEK1	0.702	4.90E-55
6	EIF4A3	0.75	1.74E-66	46	KPNB1	0.7	1.17E-54
7	SPATS2	0.744	4.67E-65	47	ORC1	0.7	1.66E-54
8	PLK1	0.742	1.53E-64	48	MYBL2	0.698	3.35E-54
9	NCAPH	0.738	1.45E-63	49	CDCA5	0.698	4.48E-54
10	PKM	0.735	8.03E-63	50	TPX2	0.697	6.52E-54
11	CCNB1	0.735	1.25E-62	51	SLC25A19	0.696	9.06E-54
12	CEP55	0.732	4.95E-62	52	KIF11	0.696	9.61E-54
13	CENPA	0.731	1.24E-61	53	STIP1	0.695	1.40E-53
14	CCDC137	0.73	1.45E-61	54	CCNB2	0.695	1.84E-53
15	NUP85	0.727	8.95E-61	55	CDK4	0.694	2.19E-53
16	G6PD	0.727	1.13E-60	56	SPC25	0.693	3.89E-53
17	BIRC5	0.725	3.15E-60	57	NUP37	0.693	3.94E-53
18	CCT2	0.723	1.06E-59	58	TPD52L2	0.692	7.23E-53
19	RAN	0.721	2.35E-59	59	CBX3	0.692	7.37E-53
20	CANT1	0.721	2.41E-59	60	CSNK1D	0.692	7.46E-53
21	KIF23	0.72	4.00E-59	61	SGO2	0.692	8.51E-53
22	PPM1G	0.72	5.12E-59	62	MCM10	0.691	9.43E-53
23	RACGAP1	0.72	5.32E-59	63	KIF18B	0.691	9.66E-53
24	CCT5	0.719	7.08E-59	64	CDCA2	0.69	1.50E-52
25	CDCA8	0.717	1.76E-58	65	WDR62	0.69	1.84E-52
26	KIF4A	0.717	2.12E-58	66	GTSE1	0.689	2.63E-52
27	BUB1B	0.717	2.46E-58	67	SSRP1	0.689	3.02E-52
28	CDC20	0.716	3.40E-58	68	TACC3	0.688	3.87E-52
29	ANLN	0.716	4.80E-58	69	POLD1	0.688	3.90E-52
30	SAE1	0.714	9.86E-58	70	KIF18A	0.688	4.00E-52
31	UTP18	0.714	1.16E-57	71	GTPBP4	0.688	4.41E-52
32	CIP2A	0.712	2.95E-57	72	GINS1	0.688	5.56E-52
33	TUBA1C	0.711	5.73E-57	73	HNRNPL	0.687	8.90E-52
34	PSMC3IP	0.71	6.90E-57	74	ERCC6L	0.686	1.28E-51
35	DLGAP5	0.708	2.28E-56	75	KIFC1	0.685	1.58E-51
36	UBE2Z	0.708	2.62E-56	76	LMNB2	0.685	2.35E-51
37	BUB1	0.707	3.41E-56	77	CWC27	0.685	2.35E-51
38	CENPO	0.707	4.29E-56	78	MELK	0.684	2.89E-51
39	SMARCD1	0.706	5.84E-56	79	ECT2	0.684	2.99E-51
40	CBX1	0.706	7.22E-56	80	MCM2	0.684	3.06E-51

No.	Gene	R	p value	No.	Gene	R	p value
81	CDC45	0.683	4.46E-51	121	RFC4	0.671	1.03E-48
82	JPT1	0.683	5.69E-51	122	MAD2L1	0.67	1.38E-48
83	NCAPD2	0.682	5.96E-51	123	UCK2	0.67	1.85E-48
84	MCM6	0.682	6.50E-51	124	PCLAF	0.67	1.92E-48
85	TCOF1	0.681	9.62E-51	125	DEPDC1B	0.67	2.01E-48
86	H2AZ1	0.681	1.10E-50	126	ENO1	0.669	2.34E-48
87	SMYD5	0.681	1.23E-50	127	BRIX1	0.669	2.62E-48
88	NUP62	0.681	1.41E-50	128	MTFR2	0.668	3.50E-48
89	CDC6	0.68	1.74E-50	129	SRRT	0.668	4.42E-48
90	KHDRBS1	0.68	1.75E-50	130	GAS2L3	0.668	4.64E-48
91	HJURP	0.68	1.80E-50	131	MCM7	0.668	4.80E-48
92	TRAIP	0.679	2.83E-50	132	CDK1	0.667	5.06E-48
93	GPN1	0.679	2.86E-50	133	EZH2	0.667	7.29E-48
94	MRPS23	0.678	4.32E-50	134	SPINDOC	0.665	1.21E-47
95	RAD54L	0.678	4.33E-50	135	DHX37	0.665	1.36E-47
96	TMEM237	0.678	4.39E-50	136	CDCA3	0.665	1.38E-47
97	CENPM	0.678	5.07E-50	137	CDT1	0.665	1.40E-47
98	SKA1	0.677	7.19E-50	138	PIGS	0.665	1.41E-47
99	ALYREF	0.677	8.85E-50	139	MCRS1	0.665	1.56E-47
100	FANCI	0.676	9.52E-50	140	FAM104A	0.665	1.62E-47
101	RBM17	0.676	9.97E-50	141	NDC80	0.665	1.79E-47
102	U2AF2	0.675	1.52E-49	142	YWHAQ	0.664	1.81E-47
103	MCM3	0.675	1.54E-49	143	FANCD2	0.664	1.86E-47
104	PA2G4	0.675	1.76E-49	144	DCTN2	0.664	1.98E-47
105	NDC1	0.675	1.77E-49	145	GNPDA1	0.664	1.99E-47
106	CEP131	0.675	1.94E-49	146	CKAP2L	0.664	2.18E-47
107	RAE1	0.675	2.23E-49	147	EME1	0.664	2.61E-47
108	GTF3C2	0.674	2.44E-49	148	CENPH	0.663	3.21E-47
109	CCT4	0.674	3.04E-49	149	DBF4B	0.663	4.21E-47
110	CSTF2	0.674	3.20E-49	150	TICRR	0.662	4.48E-47
111	SHCBP1	0.673	3.63E-49	151	MSANTD3	0.662	4.87E-47
112	DSCC1	0.673	3.66E-49	152	TOP2A	0.662	5.21E-47
113	CCT7	0.673	3.88E-49	153	COLGALT1	0.662	5.86E-47
114	ORC6	0.673	5.21E-49	154	FOXK2	0.661	7.12E-47
115	FTSJ3	0.673	5.25E-49	155	MCM4	0.661	7.28E-47
116	TTK	0.672	5.71E-49	156	FOXM1	0.661	7.40E-47
117	EXO1	0.672	5.91E-49	157	CSE1L	0.661	8.71E-47
118	TUBG1	0.672	8.08E-49	158	MSH2	0.661	9.02E-47
119	RUVBL1	0.672	8.37E-49	159	GRB2	0.66	1.39E-46
120	CHAF1B	0.671	8.80E-49	160	CCDC43	0.66	1.39E-46

No.	Gene	R	p value	No.	Gene	R	p value
161	ZWINT	0.66	1.47E-46	181	YEATS2	0.655	1.16E-45
162	FAAP24	0.66	1.47E-46	182	CPSF6	0.655	1.20E-45
163	DBF4	0.66	1.49E-46	183	MBOAT7	0.654	1.23E-45
164	ADSL	0.659	1.93E-46	184	AATF	0.654	1.47E-45
165	XRCC2	0.658	2.34E-46	185	ASF1B	0.654	1.77E-45
166	AP4M1	0.658	2.74E-46	186	DDIAS	0.653	1.91E-45
167	PKMYT1	0.658	3.03E-46	187	E2F6	0.653	2.10E-45
168	TRIM28	0.658	3.46E-46	188	ZNF207	0.653	2.57E-45
169	SFPQ	0.658	3.46E-46	189	JMJD6	0.652	3.17E-45
170	SPDL1	0.657	3.53E-46	190	UBE2T	0.652	4.01E-45
171	CAD	0.657	3.61E-46	191	DDX27	0.651	4.24E-45
172	PRC1	0.657	3.69E-46	192	RNF34	0.651	4.81E-45
173	EWSR1	0.657	4.43E-46	193	METTL6	0.65	7.46E-45
174	CENPI	0.657	5.01E-46	194	TSEN54	0.65	7.96E-45
175	NUP93	0.656	6.01E-46	195	NUF2	0.65	8.53E-45
176	ANAPC7	0.656	6.54E-46	196	FEN1	0.649	1.03E-44
177	CCNF	0.656	7.34E-46	197	TROAP	0.649	1.08E-44
178	RCC2	0.655	8.49E-46	198	GPSM2	0.649	1.17E-44
179	TPGS2	0.655	1.00E-45	199	CENPF	0.649	1.38E-44
180	RBM14	0.655	1.15E-45	200	SRP68	0.649	1.39E-44
